# Book and Pamphlet Notices

**Published:** 1858-11

**Authors:** 


### BOOK AND PAMPHLET NOTICES.

Lectures on the Principles and Practice of Physic, delivered at King's College, London. By Thomas Watson, M.D., Fellow of the Royal College of Physicians, late Physician to the Middlesex Hospital, and formerly Fellow of St. John's College, Cambridge. A new edition, from the last revised and enlarged English edition; with additions by Francis Condie, M.D., Fellow of the College of Physicians of Philadelphia, Member of the American Philosophical Society, etc., etc.; with one hundred and eighty-five Illustrations on Wood. Philadelphia: Blanchard & Lea. 1858.

"This edition of the lectures of Dr. Watson have undergone a thorough revision; and whatever of value recent research has added to our stock of knowledge in the various departments of medical science, has been carefully incorporated in them. The lectures on fever, especially, have been greatly enlarged and improved; the positive distinctions that have been insisted upon by eminent pathologists between typhus and typhoid fevers, are recognized as being founded in truth. The extent of these additions is shown by the fact that notwithstanding a very considerable enlargement in the size of the page, the work has been increased by about two hundred pages."

The above extract from the editorial preface, sets forth truthfully the main difference between this and former editions. We regard the plates for illustrating various intricate subjects treated of, as greatly increasing its value to the student.

We cannot speak too highly of this truly classical work on the practice of medicine. Take it all in all, it is the very best of books of its kind; equaled by none in beauty and elegance of diction, and not surpassed in the completeness and comprehensiveness of its contents. It will be an indispensable guide to the student in the acquirement of his profession, and no less worthy of frequent consultation and reference by the most enlightened practitioner. Dr. Condie has added to his already enviable reputation for industry and large acquirements, by the share he has taken in preparing for, and conducting through, the press, so acceptable a professional favor as this present edition of Watson's lectures.

On the Treatment of Aneurism by Compression, and by the Injection with the Perchloride of Iron. By John Reddy, M.D., Physician to the Montreal General Hospital, etc., etc.

(From the *Medical Chronicle* for September, 1858.)

The article, from which the above is the heading, is one of much interest, but does not at all correspond to its title, the principal part of it relating not to aneurism but to erectile tumor. There is, however, one case of popliteal aneurism successfully treated by compression, the report of which presents nothing unusual in similar cases.

Dr. Reddy gives a *resume* of Broca's tables, by which it appears that of 127 cases treated by compression, the deaths were not more than 5 per cent, and contrasts these with the results, as shown by Drs. Crisp and Norris, of the operations by ligature, in which it appears that the failures are about 33 per cent, and the deaths 25 per cent.

These facts speak for themselves, yet the number of cases in which compression cannot be borne, or in which it fails, is doubtless much greater than might be supposed from the tabular statements referred to.

Four cases of erectile tumor, treated by the method of Pelivay, are reported by Dr. Reddy.

The first of these was an aneurism by anastomosis, very similar in form and situation to the case first observed by John Bell, who bestowed this name upon it. Into this a solution of the perchloride of iron was injected at two different times, fifteen drops being the quantity used at the same injection.

The second case was a common erectile tumor of the size of a half crown, situated on the frontal protuberance. Into this, twelve drops were injected.

The third case was a tumor like the former in character, situated on the back. Seven drops of the solution were thrown into it.

Case four was a dark-colored nævus on the right arm, of the size of a small raspberry. Three drops of the solution were used in this case.

In all these cases the result was the same, viz: rapid solidification of the tumor, and subsequently the formation of a slough which separated in form of eschar. In the first and second cases, a portion of the tumors which escaped the action of the caustic was destroyed afterwards by the hot needles.

We chronicle these cases as showing the efforts still being made to realize the dreams of Pravaz, which, however, are becoming less frequent every year. The result was satisfactory. Dr. Reddy may well congratulate himself on having escaped the accidents which others have met with, and we cheerfully award him the credit of having proceeded with caution.

But a question necessarily arises in regard to this proceeding: is it safer or better than extirpation or the ligature? We think not, and the reasons are so obvious as not to require enumeration. John Bell extirpated in his case, and in this respect no improvement has been made in practice up to the present time.

But the result obtained by Dr. Reddy is not that sought by Pelivay, who expected by injections to coagulate the blood of aneurisms, and cause its absorption without suppuration or gangrene.

As applied in these cases, the practice may be called simply the sub-cutaneous application of caustic to erectile tumors. Dr. Brainard of this city suggested the possibility of treating tumors in this way, in a paper presented to the Society of Surgery of Paris in 1853. It did not, however, meet with a very favorable consideration in that body; and the cases reported by Dr. Reddy are the first and only instances of the adoption of a similar practice which have come within our knowledge. D. B.

We have received from the publishers:

The Physician's Visiting List, Diary and Book of Engagements, for the Year 1859. Philadelphia: Lindsay & Blackiston.

This little book is becoming, if it is not already, an "institution" among us. We notice, in addition to its other contents, that it contains Marshall Hall's ready method of resuscitation from drowning. This alone is worth the amount of the price for which it is sold, to persons who are not already perfectly familiar with it.

We have also received from the author:

A REPORT ON THE CHEMICAL ANALYSIS OF THE WHITE SULPHUR WATER OF THE ARTESIAN WELLS OF LAFAYETTE, IND.; with Remarks upon the Nature of Artesian wells. By CHARLES M. WITHERELL, Ph. D., M.D. Published by the Mayor and Council of Lafayette, at the request of a Committee of Citizens.

CASE OF CHRONIC HYDROCEPHALUS, TREATED BY INJECTIONS OF IODINE.

Dr. Tournesko, Surgeon of the Civil Hospital of Koltsa, Bucharest, has recently communicated to the *Gazette des Hopitaux* a case of hydrocephalus, treated by injection of the tincture of iodine.

The subject was a child, two months old, and the head measured about twenty inches in circumference. At the first puncture, eleven ounces of serum were drawn. This was replaced by effusion in twenty-four hours. Another puncture was made the second day, and twenty-four ounces of fluid drawn off. This time the tincture of iodine was injected (twelve *grammes* of tr. of iodine in twenty-four *grammes* distilled water), of which one-eighth part was allowed to flow out. The quantity inserted contained, according to our calculation, about sixteen grains of iodine amd three teaspoonfuls of alcohol.

At the moment of the injection, the child turned pale and cried several times. The following day he had fever, for which calomel was prescribed.

Twenty days after the operation, the head was of the natural size, and the child was in good health. Thirty-five days after the operation, the child continued in the same satisfactory state.

The following are the details of the operation:

1. A large-sized trochar was used.
2. It was carried to the depth of about two inches.
3. The puncture was made in the coronal suture, at the side (as being the point nearest the ventricles), and at an angle of forty-five degrees with the horizon.

This is the second case of hydrocephalus treated by injections of iodine. The operation was first recommended and performed by Dr. Brainard. This case, however, did not succeed, except in palliating the disease for a time. The case of Dr. Tournesko seems to have resulted more favorably, and gave more hope, at least, of a cure, although thirty-five days is too short a time to judge of the result. The only point in which the treatment differed from Dr. Brainard's was in using an alcoholic solution of iodine, in place of a watery solution with iodide of potassa.

There is one respect in which both these concern, and that is in showing the comparative innocence of iodine injections into the ventricles of the brain. This is a point concerning which a very great and important misapprehension exists. The *cerebral* substance, when cut or directly irritated, gives rise to neither pain nor spasm. This was first demonstrated by Fleurens in his great work on the nervous system, and has been confirmed by all experimenters since. The most violent poisons, such as the woorara and prussic acid, applied directly to the substance of the cerebrum, produce no effect until they are absorbed and carried into the circulation. In Dr. Brainard's case, the only severe symptoms produced (and twelve grains of iodine and thirty-six grains of iodide of potassium were injected at once and allowed to remain), occurred when the iodine, having been absorbed, was effused into the stomach, producing irritation and vomiting, as if it had been swallowed.

Considering these facts, and that a knowledge of physiology is now more common, we may hope that practitioners will not fail to give a fair trial to iodine injections in this most fatal malady, for there is no reason to believe that the so-called membrane which lines the lateral ventricles is in any respect more sensitive than the brain itself.

